# Toward a Green Revolution in soybean: The role of ultra‐high‐density planting

**DOI:** 10.1111/jipb.70079

**Published:** 2025-11-16

**Authors:** Chao Fang, Lidong Dong, Jincong Zhou, Sijia Lu, Baohui Liu

**Affiliations:** ^1^ Guangdong Key Laboratory of Plant Adaptation and Molecular Design, Guangzhou Key Laboratory of Crop Gene Editing, Innovative Center of Molecular Genetics and Evolution, School of Life Sciences Guangzhou University Guangzhou 510006 China

**Keywords:** Green Revolution, soybean, ultra‐high‐density planting

## Abstract

Soybean is a major crop that provides essential protein and oil for human consumption. Despite the increasing global demand, soybean yield has not experienced a “Green Revolution” comparable to that of rice, wheat, and maize. Here, we propose a pathway toward a soybean Green Revolution: Enhancing soybean yield through the cultivation of dwarf soybeans optimized for ultra‐high‐density planting with a dwarf and dense‐planting‐tolerant soybean variety Dongsheng 89 as a paradigmatic case. We also suggest an ideal plant architecture and specific sowing and fertilization techniques. Furthermore, we discuss the prospective application of the *rin1* gene in driving a global soybean Green Revolution, highlighting its potential to sustainably boost yields and address future food security challenges.

## Graphical abstract

A proposed soybean Green Revolution pathway aims to enhance yield through ultra‐high‐density planting by cultivating dwarf and dense‐planting‐tolerant varieties (e.g., Dongsheng 89), optimizing planting and fertilization techniques, and applying the *rin1* gene to ensure food security.

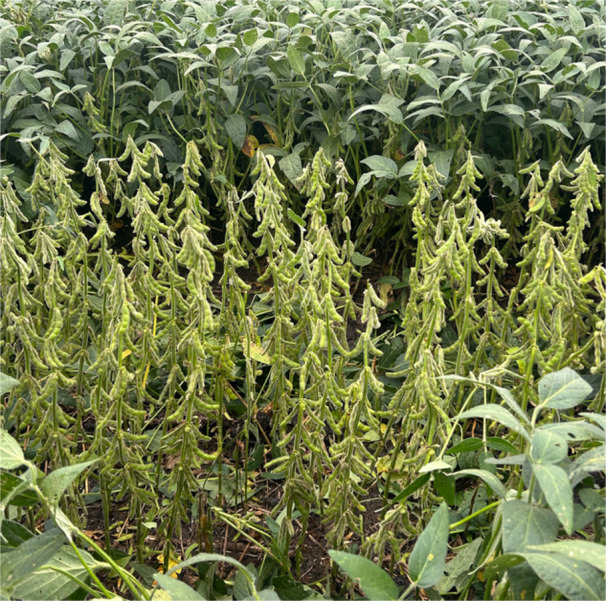

## INTRODUCTION

Soybean (*Glycine max*) is a cornerstone of global protein and oil supply. It originated in China, where it was domesticated from its wild progenitor *Glycine soja* ~5,000 years ago in the Huang‐Huai Valley of central China, a mid‐latitude region between the Yellow and Huai Rivers (Li et al., 2010; Lee et al., 2011; Sedivy et al., 2017). After domestication, soybean spread across East Asia, reaching Korea and Japan, where it became integral to local agriculture and diets. Introduced to Europe in the 17^th^ century and to the Americas by the 18^th^ century, soybean was initially grown as forage, before emerging as a major commercial crop in the 20^th^ century. Later, it was extended to South America, where Brazil and Argentina became leading producers (Hymowitz, 1970). Today, global soybean production is highly concentrated in a few major producers, with the United States, Brazil, Argentina, China, and India ranking as the top five producers (https://ourworldindata.org/). Despite its importance and widespread cultivation across many regions of the world, it has not replicated the yield breakthroughs of the Green Revolution—a pivotal 20^th^‐century agricultural transformation driven by semi‐dwarf cereal varieties (Liu Zhang et al., 2020).

Initiated in the 1960s, the Green Revolution leveraged genetic innovations (e.g., *sd1* in rice and *rht1* in wheat) that altered gibberellin biosynthesis or signaling, producing shorter, lodging‐resistant plants tolerant to high nitrogen fertilization (Hedden, 2004). However, soybean's unique biological constraints—dispersed pod distribution across stems/branches and polygenic genome complexity—have hindered similar progress (Fang et al., 2023). Although research on dwarfing caused by shortened internode length in soybeans has been reported, large‐scale production practices have not yet verified its significance for yield, nor have concrete supporting application strategies been established (Cheng et al., 2019; Chen et al., 2020, 2021; Wang et al., 2021; Xu et al., 2022; Qin et al., 2023a, 2023b; Su et al., 2024). Here, we propose a perspective that ultra‐high‐density planting is a viable pathway toward achieving a Green Revolution of soybean, supported by empirical evidence from the dwarf and dense‐planting‐tolerant soybean variety Dongsheng 89, which is capable of adapting to ultra‐high‐density planting (nearly twice the planting density of conventional varieties). Additionally, we propose an ideal plant architecture and corresponding seeding strategies, and look forward to the future of the soybean Green Revolution.

### The Green Revolution and its absence in soybeans

Globally, soybeans are considered one of the four major food crops—along with rice, wheat, and maize—that are essential to human nutrition and food security (Iizumi and Sakai, 2020). However, unlike cereals, soybean has experienced much slower progress in both total production and yield improvement over the past half century, largely because it did not benefit from the Green Revolution. In cereals, intensive nitrogen fertilization was once considered essential for yield gains, but these practices frequently led to severe lodging and associated yield loss, limiting productivity (Crook and Ennos, 1995). A breakthrough emerged with the development of semi‐dwarf rice and wheat varieties by scientists and breeders. Compared with traditional cultivars, these lines displayed stronger lodging resistance, stable performance under dense planting, and tolerance to much higher fertilizer input. Their widespread adoption effectively overcame the limitations imposed by excessive fertilization and high planting density, enabling a substantial increase in yield per unit area (Hedden, 2004). The combination of semi‐dwarf varieties with improved fertilizer management, irrigations, mechanization, and crop protection technologies triggered unprecedented yield gains in cereals. This transformation not only revolutionized agricultural productivity but also provided critical support for global food security in the latter half of the 20^th^ century (Evenson and Gollin, 2003). This period is collectively known as the “Green Revolution” (Hedden, 2004).

Subsequent molecular studies revealed that the Green Revolution is largely driven by modifications in gibberellin (GA) biosynthesis and signaling. In rice, the *sd1* gene encodes a key enzyme in the gibberellin biosynthesis (Monna et al., 2002; Sasaki et al., 2002), while in wheat, the *Rht1* gene encodes a DELLA protein that suppresses gibberellin signaling (Peng et al., 1999). These genetic changes produced semi‐dwarf phenotypes with enhanced lodging resistance, greater tolerance to nitrogen fertilization, and yield stability under high planting density. Consequently, semi‐dwarf and high‐input‐tolerant varieties remain the foundation of rice and wheat breeding programs, and their adoption has sustained substantial yield gains in cereals since the 1960s.

**Figure 1:**
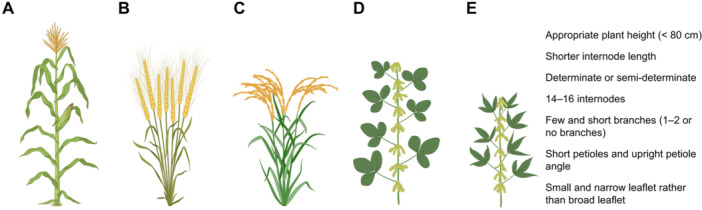
Plant architecture of major crop **(A–D)** Seed distribution of major crops. Seed distribution in maize **(A),** wheat **(B)**, rice **(C)**, and soybean **(D)**. **(E)** Ideal plant architecture for ultra‐high‐density planting of soybeans.

However, such dramatic yield gains and the utilization of dwarfing traits have not been achieved in soybeans (Liu Zhang et al., 2020). A major limitation arises from its pod‐bearing architecture. In cereals, grains are concentrated at the top of the panicle, and dwarfing allows increased yield without substantially reducing grain number per plant. By contrast, soybean pods are distributed across multiple nodes along the main stem and branches. Simple reductions in plant height therefore risk decreasing the total number of pods per plant, offsetting potential yield benefits (Fang et al., 2023) (Figure 1A–D). Moreover, soybeans possess a symbiotic nitrogen fixation system, with more than 60% of their nitrogen supply derived from root nodules. Excessive nitrogen fertilization can impair the nitrogen fixation efficiency of soybeans (Su et al., 2023). As a result, the yield‐enhancing effect of nitrogen fertilization on soybeans is relatively smaller compared to other crops, and the need for dwarfing traits to mitigate lodging caused by excessive nitrogen is also less pronounced in soybeans. Therefore, since the domestication of soybeans and the widespread use of chemical fertilizers, dwarfing traits have not been a primary focus in soybean breeding, and consequently, dwarf germplasm resources remain relatively scarce.

Besides architectural constraints, soybean's paleotetraploidy origin has endowed it with an exceptionally complex genome characterized by extensive gene duplication (Schmutz et al., 2010). Most agronomic traits are governed by a polygenic network or large gene families, making both genetic improvement and mechanistic dissection highly challenging. This genomic redundancy complicates the identification of single major‐effect genes capable of producing dramatic phenotypic shifts—analogous to the “Green Revolution genes” in rice and wheat. Consequently, progress in soybean yield improvement has lagged behind that of cereals, underscoring the need for novel breeding paradigms to overcome these intrinsic biological barriers.

### Dwarf varieties and ultra‐high‐density planting represent the pathway to a Green Revolution in soybeans

Here, we put forward a perspective to breed dwarf soybean varieties with improved tolerance to dense planting, which may even enable soybeans to withstand ultra‐high planting densities (no less than 450,000 plants/ha), thereby contributing to yield enhancement. High‐density planting enables soybean to form a relatively closed canopy, improving light interception and reducing bare soil and weed competition. Although individual plants receive less light, the overall light use efficiency of the population increases, leading to greater total photosynthetic products. Under high‐density planting, individual plant competition intensifies, branching decreases, and more nutrients are allocated to the main stem and pod‐setting areas, which favors an increase in the pod‐setting rate. High‐density planting can also alter plant architecture and adjust the allocation of nutrients to various plant parts, especially between the above‐ground parts and the root nodules. However, the shading effects induced by high‐density planting may cause excessive elongation and increase the risk of lodging. Therefore, utilizing dwarfing traits to improve soybean tolerance to high‐density planting and leveraging the population effect to enhance soybean yield represent a viable approach in the soybean Green Revolution.

In the process of validating this perspective and implementing this approach, to address the scarcity of dwarf resources in soybeans, we generated a mutant library through γ‐irradiation of the cultivar Heinong 35 in 2011 and identified a dwarf mutant, *rin1* (*reduced internode 1*). After years of selection, the variety Dongsheng 89 was officially registered in 2019. Dongsheng 89 has a semi‐dwarf, erect architecture (~70 cm height at a density of 45,000 plants/ha, 14–16 main stem nodes, 1–2 branches), with a semi‐determinate podding habit, purple flowers, pointed leaves, and uniform pod distribution. It has a 100‐seed weight of ~16–18 g, seed protein and oil contents of 42.05% and 20.80%, respectively, and an early‐maturing growth period of ~115 d (Li et al., 2023), requiring ≥ 2,300°C active accumulated temperature (≥ 10°C basis). It is well suited for cultivation in the third accumulated temperature zone of Heilongjiang Province. Subsequent study has demonstrated that *RIN1* encodes a homolog of Arabidopsis SUPPRESSOR OF PHYA 3 (SPA3). RIN1 protein interacts with STF1/STF2 (homologs of ELONGATED HYPOCOTYL 5 in Arabidopsis) and promotes their protein degradation, thereby suppressing the STF1/STF2‐mediated induction of GA2ox7a/GA2ox7b. As a consequence, the endogenous level of gibberellin GA_1_ is elevated, ultimately regulating soybean internode length (Li et al., 2023).

From 2022 to 2024, large‐scale field (no less than five hectares) demonstrations in Hailun (47°52′ N, 126°58′ E, the Fourth Accumulated Temperature Zone of Heilongjiang Province) recorded yields of 3,854.9 kg/ha at a planting density of 400,000 plants/ha, 4,059.0 kg/ha at 450,000 plants/ha, and 4,063.5 kg/ha at 450,000 plants/ha.

**Figure 2:**
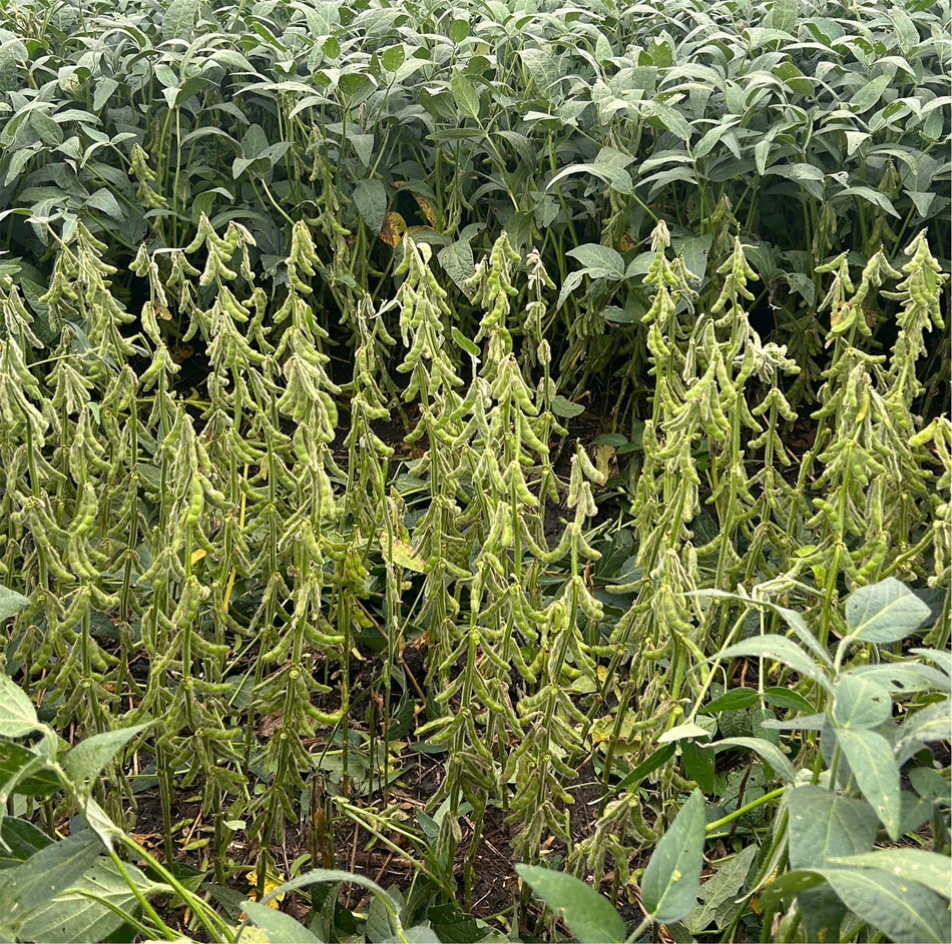
Field photo of Dongsheng 89 The petioles and leaves of the plants in the foreground have been removed to allow for an unobstructed view of the planting density.

In 2025, due to the lodging resistance and high‐density planting adaptability of Dongsheng 89, it was planted at an ultra‐high density of 550,000 plants/ha over 770 mu (~51.3 ha) in Donghe Village, Dongsheng Township, Beian City, Heilongjiang Province (48°28′ N, 126°79′ E, the Fourth Accumulated Temperature Zone of Heilongjiang Province) (Figure 2). Yield measurements showed a survival rate of 520,000 plants/ha, with a yield of 5,795.25 kg/ha. This resulted in a significant increase in both the planting density and the per‐hectare yield of soybeans. These values are nearly three times the national average soybean yield in China (~1,998 kg/ha) at the conventional planting density of 250,000 to 300,000 plants/ha in 2023–2024 (https://www.stats.gov.cn/).

### The ideal plant architecture and specific sowing and fertilization techniques for ultra‐high‐density soybean cultivation

The concept of an ideal soybean architecture has already been proposed (Liu Zhang et al., 2020; Li et al., 2025). Drawing on the performance of Dongsheng 89, we put forward a new model of the ideal soybean plant type—particularly one adapted to ultra‐high‐density planting (Figure 1E):

1. *Plant height and internode traits*: Under high‐density planting conditions, plants should maintain a height of < 80 cm with shortened internodes, thick and rigid stems, and strong lodging resistance.

2. *Branching and canopy architecture*: The overall plant architecture should be compact, featuring few and short branches (1–2 or no branches) with small angles relative to the main stem. Pod formation should occur predominantly along the main stem to minimize inter‐plant shading.

3. *Reproductive habit*: Varieties with determinate or semi‐determinate habits are preferred, ensuring that pods are concentrated and uniformly distributed on the main stem. This reduces excessive canopy closure associated with highly branched types.

4. *Node and pod traits*: Having 14–16 nodes, each densely set with pods, is essential. An increase in pod number per plant is expected to scale positively with planting density, thereby compensating for the reduced plant size and supporting yield gains at the population level.

5. *Leaf morphology and dynamics*: The plant has a tower‐like structure. The leaves are narrow‐leaflet rather than broad‐leaflet, and relatively small. This arrangement optimizes the vertical light penetration within the canopy, reducing mutual shading, and maximizes light capture.

Moreover, appropriate sowing and fertilization techniques are essential components of high‐density planting. Traditional ridge planting is inadequate for ultra‐high‐density planting exceeding 45 plants/ha, necessitating the adoption of flat sowing techniques. To address the issue of insufficient soil temperature resulting from broadcasting, the application of organic fertilizers becomes imperative.

### Concluding remarks and future perspectives

The “Green Revolution” of the 1960s, driven by the deployment of dwarfing genes, successfully overcame lodging and yield losses associated with intensive fertilizer use, thereby revolutionizing global rice and wheat production (Hedden, 2004). However, soybean—despite its status as a major source of plant‐derived protein and vegetable oil—has not undergone a comparable yield breakthrough. This disparity highlights the pressing need for a soybean “Green Revolution.”

Recently, we developed a concept for increasing soybean yield through flat planting with ultra‐high‐density planting (no less than 450,000 plants/ha) of dwarf, lodging‐resistant soybeans. As a practical example, we used Dongsheng 89, which can be planted at twice the conventional soybean planting density and achieves extremely high yields in the Fourth Accumulated Temperature Zone of Heilongjiang Province. Guided by this concept, introduction of molecular modules like *rin1* with short internodes through conventional breeding or precise genome editing methods, and combined with ultra‐high‐density planting, could significantly increase the average soybean yield in China and even on a global scale. Beyond single‐gene utilization, integration of *rin1* with optimized plant architecture, ultra‐high‐density management practices, and advanced genomic breeding strategies could lay the foundation for a paradigm shift in soybean improvement. Collectively, these advances may ultimately catalyze a long‐awaited Green Revolution in soybean production, providing a robust pathway to strengthen global food and nutritional security.

### Generative AI usage statement

During the preparation of this work, the authors utilized DeepSeek for the purpose of polishing the text and refining the nuances of the language. After using this tool, the authors carefully reviewed and revised the content where necessary, and take full responsibility for the final version of the publication.

## CONFLICTS OF INTEREST

The authors of this work have no conflicts of interest to declare.

## AUTHOR CONTRIBUTIONS

C.F. and L.D. drafted the manuscript. J.Z., S.L., and B.L. conceived the article and revised the manuscript. All authors have read and approved the contents of this paper.
